# Quantitative Mass Spectrometry Analysis of Cerebrospinal Fluid Protein Biomarkers in Alzheimer’s Disease

**DOI:** 10.1038/s41597-023-02158-3

**Published:** 2023-05-09

**Authors:** Caroline M. Watson, Eric B. Dammer, Lingyan Ping, Duc M. Duong, Erica Modeste, E. Kathleen Carter, Erik C. B. Johnson, Allan I. Levey, James J. Lah, Blaine R. Roberts, Nicholas T. Seyfried

**Affiliations:** 1 Department of Neurology, Emory University School of Medicine, Atlanta, USA; 2 Department of Biochemistry, Emory University School of Medicine, Atlanta, USA

**Keywords:** Diagnostic markers, Alzheimer's disease, Cerebrospinal fluid proteins

## Abstract

Alzheimer’s disease (AD) is the most common form of dementia, with cerebrospinal fluid (CSF) β-amyloid (Aβ), total Tau, and phosphorylated Tau (pTau) providing the most sensitive and specific biomarkers for diagnosis. However, these diagnostic biomarkers do not reflect the complex changes in AD brain beyond amyloid (A) and Tau (T) pathologies. Here, we report a selected reaction monitoring mass spectrometry (SRM-MS) method with isotopically labeled standards for relative protein quantification in CSF. Biomarker positive (AT+) and negative (AT−) CSF pools were used as quality controls (QCs) to assess assay precision. We detected 62 peptides (51 proteins) with an average coefficient of variation (CV) of ~13% across 30 QCs and 133 controls (cognitively normal, AT−), 127 asymptomatic (cognitively normal, AT+) and 130 symptomatic AD (cognitively impaired, AT+). Proteins that could distinguish AT+ from AT− individuals included SMOC1, GDA, 14-3-3 proteins, and those involved in glycolysis. Proteins that could distinguish cognitive impairment were mainly neuronal proteins (VGF, NPTX2, NPTXR, and SCG2). This demonstrates the utility of SRM-MS to quantify CSF protein biomarkers across stages of AD.

## Background & Summary

Alzheimer’s disease (AD) affects more than 45 million people worldwide, making it the most common neurodegenerative disease^1–3^. AD biomarker research has predominately focused on β-amyloid (Aβ) and Tau, as these proteins reflect pathological Aβ plaques and tau neurofibrillary tangles (NFT), respectively, in AD^4,5^. Although Aβ and Tau are the most sensitive and specific CSF biomarkers for diagnosis^6,7^ these two proteins do not reflect the heterogenous and complex changes in AD brain^8,9^. Furthermore, failed clinical trials of Aβ-based therapeutic approaches highlight the complexity of AD and the need for additional biomarkers to fully illustrate pathophysiology for advancements in diagnostic profiling, disease monitoring, and treatments^1–3,9^.

Considering the diagnostic challenges related to the overlapping pathologies of neurodegenerative diseases, AD biomarkers that represent diverse pathophysiological changes could facilitate an early diagnosis, predict disease progression, and enhance the understanding of neuropathological changes in AD^1^. AD has a characteristic pre-clinical or asymptomatic period (AsymAD) where individuals have AD neuropathology in the absence of clinical cognitive decline^5,10,11^. Thus, biomarkers for the prodromal phase of AD that can begin changing years or decades before signs of cognitive impairment, would be valuable for disease intervention, clinical trial stratification, and monitoring drug efficacy.

Proteins are the proximate mediators of disease, integrating the effects of genetic, epigenetic, and environmental factors^9,12^. Network proteomic analysis has emerged as a valuable tool for organizing complex unbiased proteomic data into groups or “modules” of co-expressed proteins that reflect various biological functions, i.e., systems biology^13–16^. The direct proximity of CSF to the brain presents a strong rationale to integrate the brain and CSF proteomes to increase the pathophysiological diversity among biofluid biomarkers of AD^7,17^. We recently integrated a human AD brain proteomic network with a CSF proteome differential expression analysis and revealed approximately 70% of the CSF proteome overlapped with the brain proteome^18^. Nearly 300 CSF proteins were identified as significantly altered between control and AD samples, representing predominately neuronal, glial, vasculature, and metabolic pathways, creating an excellent list of candidates for further quantification and validation.

Here, we developed a high-throughput targeted selected reaction monitoring-based mass spectrometry (SRM-MS) assay^19^ to quantify and validate reliably detected CSF proteins in healthy individuals and individuals with asymptomatic or symptomatic AD for staging AD progression. We evaluated 200+ tryptic peptides that were selected using a data-driven approach from the integrated brain-CSF proteome network analysis. We selected peptides with differential abundance in AD CSF observed in >50 percent of case samples by discovery proteomics^18^ for synthesis as crude heavy standards. We used two pooled CSF reference standards to determine which peptides were reliably detected in CSF matrix. We reproducibly detected and reliably quantified 62 tryptic peptides from 51 proteins in 390 clinical samples and 30 pooled reference standards. Furthermore, using a combination of differential expression and receiver operating curve (ROC) analyses we found CSF proteins that can best discriminate stages of AD progression. Collectively, these data highlight the utility of a high throughput SRM-MS approach to quantify biomarkers associated with AD that ultimately hold promise for monitoring disease progression, stratifying patients for clinical trials, and measuring therapeutic response. Future studies will be necessary to assess the diagnostic and predictive utility of our CSF peptide SRM panel against gold-standard CSF (amyloid, tau and pTau) and imaging AD biomarkers in larger prospective patient cohorts.

## Methods

### Reagents and materials

Heavy labeled peptides (Thermo PEPotec SRM Peptide Libraries; Grade 2; crude as synthesized), trypsin, mass spectrometry grade, trifluoroacetic acid (TFA), foil heat seals (AB-0757), and low-profile square storage plates (AB-1127) were purchased from Thermo Fisher Scientific (Waltham, MA). Lysyl endopeptidase (Lys-C), mass spectrometry grade was bought from Wako (Japan); sodium deoxycholate, CAA (chloroacetamide), TCEP (tris-2(-carboxyethyl)-phosphine), and triethylammonium hydrogen carbonate buffer (TEAB) (1 M, pH 8.5) were obtained from Sigma (St. Louis, MO). Formic acid (FA), 0.1% FA in acetonitrile, 0.1% FA in water, methanol, and sample preparation V-bottom plates (Greiner Bio-One 96-well Polypropylene Microplates; 651261) are from Fisher Scientific (Pittsburgh, PA). Oasis PRiME HLB 96-well, 30 mg sorbent per well, solid phase extraction (SPE) cleanup plates were from Waters Corporation (Milford, MA).

### CSF collection and immunoassay measurements

Specimen collection was accomplished under several separate Institutional Review Board (IRB) protocols reviewed by the Emory University Institutional Review Board (IRB00024959, IRB00078273, IRB00079069, and IRB00080300), each of which included signed informed consent allowing for broad sharing of specimens. The work reported here, which constitutes secondary use of existing data/specimens, was also reviewed, and approved by the Emory Institutional Review Board (STUDY00001741). CSF was collected by lumbar puncture and banked according to 2014 ADC/NIA best practices guidelines https://www.alz.washington.edu/BiospecimenTaskForce.html. CSF samples from all participants were collected in a standardized fashion applying common preanalytical methods. Emory Healthy Brain Study (EHBS)^20^ participants were asked to fast for at least 6 hours prior to lumbar puncture (LP) procedures and CSF collection. All clinicians performing LPs in the Cognitive Neurology Clinic are also active investigators in the EHBS and apply shared standard work in both settings. LPs are performed using a 24 g atraumatic Sprotte spinal needle (Pajunk Medical Systems, Norcross, GA) with aspiration and, after clearing any blood contamination, CSF is transferred from syringe to 15 ml polypropylene tubes (Corning, Glendale, AZ), which are inverted several times. The CSF (0.5 mL) is aliquoted without further handling into 0.9 mL FluidX tubes (Azenta, Chemsford, MA) and placed into dry ice/methanol bath prior to transfer to −80 °C freezers. Time from initial collection to storage at −80 °C is less than 60 minutes. Aβ42, tTau, and pTau assays were performed on CSF samples following a single freeze-thaw cycle on a Roche Cobas e601 analyzer using the Elecsys assay platform^21^. All assays were performed in a single laboratory in the Emory Goizueta Alzheimer’s Clinical Research Unit following manufacturer’s recommended protocols.

### Pooled CSF as quality controls

Two pools of CSF were generated based on Aβ(1–42), total Tau, and pTau181 levels to create AD-positive (AT+) and AD-negative (AT−) quality control standards. Each pool consisted of approximately 50 mL of CSF by combining equal volumes of CSF selected from well-characterized samples (~45 unique individuals per pool) from the Emory Goizueta Alzheimer’s Disease Research Center (GADRC) and EHBS^20^. AD biomarker status for individual cases was determined on the Roche Elecsys® immunoassay platform^21–23^; the average CSF biomarker value is reported in parentheses. The control CSF pool (AT−) was comprised of cases with relatively high levels of Aβ(1–42) (1457.3 pg/mL) and low total Tau (172.0 pg/mL) and pTau181 (15.1 pg/mL). In contrast, the AD pool (AT+) was comprised of cases with low levels of Aβ(1–42) (482.6 pg/mL) and high total Tau (341.3 pg/mL) and pTau181 (33.1 pg/mL). The quality control (QC) pools were processed and analyzed identically to the CSF clinical samples reported.

### Clinical characteristics of the cohort

**Fig. 1:**
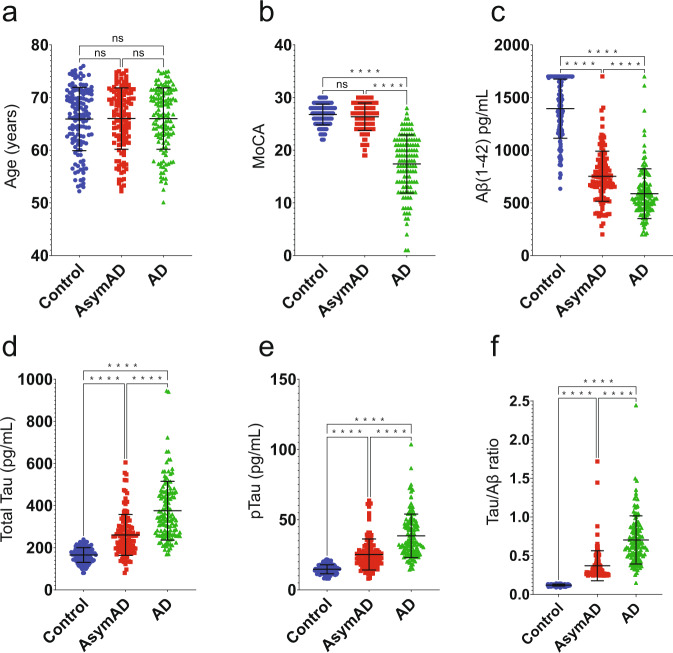
Cohort characteristics. A total of 390 samples (133 controls, 127 AsymAD, 130 AD unless otherwise noted) were analyzed using the following characteristics for grouping. (**a**) Age range across each group of the cohort was carefully selected to balance for age and sex (Table 1). (**b**) Cognition was assessed using the Montreal Cognitive Assessment (MoCA) score; there is no significant difference in scores between the Control and AsymAD groups serving as the two cognitively normal diagnostic groups (133 controls, 127 AsymAD, 124 AD). The Roche Diagnostics Elecsys® platform was used for CSF biomarker measurements for Aβ(1–42) (**c**), Total Tau (133 controls, 127 AsymAD, 129 AD) (**d**), and pTau (**e**) (pg/mL) showing the significance between groups for each measurement. (**f**) Tau/Aβ ratio data across control, AsymAD, and AD groups (133 controls, 127 AsymAD, 129 AD). The significance of the pairwise comparisons is indicated by overlain annotation of ‘ns’ (not significant; p > 0.05) or asterisks; ****p ≤ 0.0001.

Human cerebrospinal fluid (CSF) samples from 390 individuals including 133 healthy controls, 130 patients with symptomatic AD, and 127 asymptomatic AD patients (cognitively normal but AD biomarker positive) were obtained from Emory’s GADRC and EHBS (Fig. 1 and Table 1). All symptomatic individuals were diagnosed by expert clinicians in the ADRC and Emory Cognitive Neurology Program, who are subspecialty trained in Cognitive and Behavioral Neurology, following extensive clinical evaluations including detailed cognitive testing, neuroimaging, and laboratory studies. CSF samples were selected to balance for age and sex (Table 1). For biomarker measurements, CSF samples from all individuals were assayed for Aβ(1–42), total Tau, and pTau using the Roche Diagnostics Elecsys® immunoassay platform^21–23^. The cohort characteristics are summarized in Fig. 1 and Table 1. Compared to our previous CSF studies^16,18,24^, there is minimal overlap with 329 of the 390 CSF samples (~84%) unique to this study. Samples were stratified into controls, AsymAD, and AD based on Tau and Amyloid biomarkers status and cognitive score from the Montreal Cognitive Assessment (MoCA). All case metadata including disease state, age, sex, race, apolipoprotein (ApoE) genotype, MoCA scores, and biomarkers measurements were deposited on Synapse^25^.

**Table 1:** Cohort Characteristics.

Sample Group	CT	AsymAD	AD
Sample Size	N = 133	N = 127	N = 130
**Characteristics**			
Sex	99 F, 34 M	94 F, 33 M	97 F, 33 M
Age^a^	66 ± 6	66 ± 6	66 ± 6
MoCA^b^	26.8 ± 2.0	26.4 ± 2.6	17.4 ± 5.5
CSF Aβ_42_^b^	1394.0 ± 280.7	752.0 ± 238.2	587.0 ± 236.8
CSF tTau^b^	165.8 ± 35.0	260.8 ± 97.2	375.7 ± 139.6
CSF pTau^b^	14.7 ± 3.2	25.2 ± 11.1	38.5 ± 15.5

^a^Age in years. Values given as average ± standard deviation.

^b^MoCA, CSF Aβ_42_, CSF tTau, and CSF pTau in pg/mL. Values given in average ± standard deviation.

Abbreviations: CT, Control; AD, Alzheimer’s disease; MoCA, Montreal Cognitive Assessment.

### Peptide selection and selected reaction monitoring assay

We harnessed both deep discovery and single-shot tandem mass tag (ssTMT) peptide data from CSF proteomics^16,18^. Here, we prioritized peptides for SRM validation that i) had one or more spectral match, ii) were differentially abundant (AD versus control) iii) or that mapped to proteins within brain-based biological panels that differed in AD^18^. Ultimately, we nominated 200+ peptides for synthesis as crude heavy standards. The heavy peptides contained isotopically labeled C-terminal lysine or arginine residues (^13^C, ^15^N) for each tryptic peptide. Based on the crude heavy peptide signal, the peptides were pooled to achieve total area signals ≥1 × 10^5^ in CSF matrix. The transition lists were created in Skyline-daily software (version 21.2.1.455)^26,27^. An in-house spectral library was created in Skyline based on tandem mass spectra from CSF samples. Skyline parameters were specified as: trypsin enzyme, Swiss-Prot background proteome, and carbamidomethylation of cysteine residues (+57.02146 Da) as fixed modifications. Isotope modifications included: ^13^C_6_^15^N_4_ (C-term R) and ^13^C_6_^15^N_2_ (C-term K). The top ten fragment ions that matched the criteria (precursor charges: 2; ion charges 1, 2; ion types: y, b) were selected for scrutiny. The top 5–7 transitions per heavy precursor were selected by manual inspection of the data in Skyline and scheduled transition lists were created for collision energy optimization. Collision energies were optimized for each transition; the collision energy was ramped around the predicted value in 3 steps on both sides, in 2 V increments^28^. The selected transitions were tested in real matrix spiked with heavy peptide mixtures. The three best transitions per precursor were selected by manual inspection of the data in Skyline and one scheduled transition list was created for the final assays. A list of transitions used in this study was deposited on Synapse^25^.

### Preparation of CSF for mass spectrometric analysis

All CSF samples were blinded and randomized. Each CSF sample was thawed and aliquoted into sample preparation V-bottom plates that also included quality controls. Each sample and quality control were processed independently in parallel. Crude CSF (50 µL) was reduced, alkylated, and denatured with tris-2(-carboxyethyl)-phosphine (TCEP, 5 mM), chloroacetamide (CAA, 40 mM), and sodium deoxycholate (1%) in triethylammonium bicarbonate buffer (TEAB, 100 mM) in a final volume of 150 µL. Sample plates were heated at 95 °C for 10 min, followed by a 10-min cool down at room temperature while shaking on an orbital shaker (300 rpm)^29^. CSF proteins were digested with Lys-C (Wako; 0.5 µg; 1:100 enzyme to CSF volume) and trypsin (Pierce; 5 µg; 1:10 enzyme to CSF volume) overnight in a 37 °C oven. After digestion, heavy labeled standards for relative quantification (15 µL per 50 µL CSF) were added to the peptide solutions followed by acidification to a final concentration of 0.2% TFA and 2% FA (pH ≤ 2). Sample plates were placed on an orbital shaker (300 rpm) for at least 10 minutes to ensure proper mixing. Plates were centrifuged (4680 rpm) for 30 minutes to pellet the precipitated surfactant. Peptides were desalted with Oasis PRiME HLB 96-well, 30 mg sorbent per well, solid phase extraction (SPE) cleanup plates from Waters Corporation (Milford, MA) using a positive pressure system. Each SPE well was conditioned (500 µL methanol) and equilibrated twice (500 µL 0.1% TFA) before 500 µL 0.1% TFA and supernatant were added. Each well was washed twice (500 µL 0.1% TFA) and eluted twice (100 µL 50% acetonitrile/0.1% formic acid). All eluates were dried under centrifugal vacuum and reconstituted in 50 µL mobile phase A (0.1% FA in water) containing Promega 6 × 5 LC-MS/MS Peptide Reference Mix (50 fmol/µL; Promega V7491).

### Liquid chromatography-tandem mass spectrometry (LC-MS/MS)

Peptides were analyzed using a TSQ Altis Triple Quadrupole mass spectrometer (Thermo Fisher Scientific). Each sample was injected (20 μL) using a 1290 Infinity II system (Agilent) and separated on an AdvanceBio Peptide Map Guard column (2.1 × 5 mm, 2.7 μm, Agilent) connected to AdvanceBio Peptide Mapping analytical column (2.1 × 150 mm, 2.7 μm, Agilent). Sample elution was performed over a 14-min gradient using mobile phase A (MPA; 0.1% FA in water) and mobile phase B (MPB; 0.1% FA in acetonitrile) with flow rate at 0.4 mL/min. The gradient was from 2% to 24% MPB over 12.1 minutes, then from 24% to 80% over 0.2 min and held at 80% B for 0.7 min. The mass spectrometer was set to acquire data in positive-ion mode using selected reaction monitoring (SRM) acquisition. Positive ion spray voltage was set to 3500 V for the Heated ESI source. The ion transfer tube and vaporizer temperatures were set to 325 °C and 375 °C, respectively. SRM transitions were acquired at Q1 resolution 0.7 FWHM, Q2 resolution 1.2 FWHM, CID gas 1.5 mTorr, 0.8 s cycle time.

### Data analysis

Raw files from Altis TSQ were uploaded to Skyline-daily software (version 21.2.1.455), which was used for peak integration and quantification by peptide ratios. QC SRM data were manually evaluated in Skyline by assessing retention time reproducibility, matching light and heavy transitions using Ratio Dot Product, and determining the peptide ratio precision using coefficient of variation (CV) by QC condition. If Skyline could not automatically pick a consistent peak due to interference in the light transitions the peptide was removed from the analysis. Transition profiles were checked to insure the heavy and light transition profiles matched using the Ratio Dot Product value in Skyline. The Ratio Dot Product (1 = exact match) is a measure of whether the transition peak areas in the two label types are in the same ratio to each other. The average Ratio Dot Product value for each peptide was >0.90 for each QC. If the retention time or Ratio Dot Product were outside of the expected range for a peptide in a few samples, the peaks were checked individually and adjusted as necessary. Total area ratios for each peptide were calculated in Skyline by summing the area for each light (3) and heavy (3) transition and dividing the light total area by the heavy total area. The Total Area Ratio CV was assessed using Skyline and the peptide was removed from the analysis if the CV > 20% by QC condition. Next, the individual CSF samples were analyzed in a blind fashion. We used the total area ratios (peptide ratios) for each targeted peptide in each sample and QC analysis. The Data Matrix is a table of peptide ratios without imputation. The data matrix does not contain blank cells or missing data; however, there are zero measures for the APOE2 allele-specific peptide because it is not present in those samples (reviewed manually) due to genetic background. The raw data files and Skyline file were deposited on Synapse^25^.

### Statistical analyses

We used Skyline-daily software (version 21.2.1.455) and GraphPad Prism (version 9.4.1) software to calculate means, medians, standard deviations, and coefficients of variations^27^. Peptide abundance ratios were log2-transformed. Since log2 of zero is undefined we imputed the zero values as one-half the minimum nonzero ratio measurement so that the APOE2 allele-specific peptide would not contain undefined values. Then, one-way ANOVA with Tukey *post hoc* tests for significance of the paired groupwise differences across diagnosis groups was performed in R (version 4.0.2) using a custom calculation and volcano plotting framework implemented and available as an open-source set of R functions documented further on https://www.github.com/edammer/parANOVA. T test p values and Benjamini-Hochberg FDR for these are reported for two total group comparisons, as was the case for AT+ versus AT− peptide mean difference significance calculations. Receiver-operating characteristic (ROC) analysis was performed in R (version 4.0.2) with a generalized linear model binomial fit of each set of peptide ratio measurements to the binary case diagnosis subsets AD/Control, AsymAD/Control, and AD/AsymAD using the pROC package implementing ROC curve plots, and calculations of AUC and AUC DeLong 95% confidence interval. Additional ROC curve characteristics including sensitivity, specificity, and accuracy were calculated with the reportROC R package. Robustness of the ROC calculations of AUC were confirmed using k-fold cross-validation (k = 10 folds, with each fold containing case subsets with equal distributions of the binary outcome) implemented using the cvAUC R package functions for calculating cross-validated AUC (cvAUC), and confidence interval on pooled predictions, and these calculations were consistently within 1 percent of AUC as calculated using a single calculation on the full data (data not shown). Venn diagrams were generated using the R vennEuler package, and the heatmap was produced using the R pheatmap package/function. R boxplot function output was overlaid with beeswarm-positioned individual measurement points using the R beeswarm package. Pearson correlations of SRM peptide measurements to immunoassay measurements of Aβ(1–42), total Tau, phospho-T181 Tau, and the ratio of total Tau/Aβ were performed using the corAndPvalue WGCNA function in R. Correlation scatterplots were generated using the verboseScatterplot WGCNA function.

## Data Records

Raw data and transition list are available on PeptideAtlas (PASS038140)^30^. All files pertaining to this manuscript have been deposited on Synapse^25^ and the Emory AD CSF SRM project folder structure and contents are described as:

1. Analysis folder contains the R files and inputs used to create the volcano plots and ROC curves. Comments describing the R files can be found at https://www.github.com/edammer/parANOVA.
2. Data folder
  - ANOVA file contains the full statistics from the ANOVA and Tukey post-hoc analysis.
  - ELISA_PearsonCors file contains the Pearson correlations (rho), Student p values of correlation significance, and numbers of paired observations for correlation of biomarker peptide abundances to immunoassay measures of Aβ(1–42), total Tau, phospho-T181 Tau, and the ratio of total Tau/Aβ.
  - EmoryMethodsReport document is a brief description of the methods and data set.
  - PeptideRatiosDataMatrix spreadsheet contains light to heavy ratio for each peptide in each sample. The 62 peptides are listed in the first column. The individual samples are shown by file name across the top row with AT+ QC samples beginning with AD_, AT− QC samples beginning with CTL_, and individual samples mapping back to the Metadata using the sample run order (Sxxx) or SampleID (_xxxxx). Plate/box and well position are also defined in the file name (_Bx_Axx).
  - PeptideTransitions spreadsheet contains protein, protein preferred name, protein gene, peptide sequence, modified peptide sequence, isotope label, precursor m/z, precursor charge, product m/z, collision energy, and fragment ion.
  - ProteinDetails spreadsheet lists the protein, description, accession, preferred name, and gene.
  - QCstatistics file contains the QC statistics for the biomarker and APOE allele-specific peptides including CV as percent, mean, median, and ratio dot product values for the peptide ratios.
  - ROCstatistiscs file contains the ROC curve statistics including AUC, p, 95% DeLong confidence interval, accuracy, specificity, and sensitivity for dichotomous diagnosis case sample groups.
  - SampleMetadata spreadsheet contains the available traits for each sample analyzed including:
    i) SampleID – Internal Sample Identifier
    ii) Age(years) – Deidentified Age in years
    iii) Sex – Binary Sex
    iv) Race – self-identified race
    v) Educ – formal years of education (years)
    vi) MoCA – Montreal Cognitive Assessment Score ranging from 0–30
    vii) APOE status – APOE genotype
    viii) Aβ42, tTau, pTau – as measured in CSF by Roche Elecsys® immunoassay platform (pg/mL)
    ix) tTau:Aβ42 – ratio of tTau/Aβ42
    x) SampleRunOrder – the order the samples were acquired
    xi) Condition – the Control/AsymAD/AD group used in the analysis
3. The MS RAW files folder contains all mass spectrometry raw files (N = 423) from both quality control replicates and clinical samples.
4. The Skyline quantification folder contains:
  - SkylineData62peps file that was used to report the peptide ratios, calculate CVs and means, and ratio dot product.
  - SkylineAcquiredData245peptidesPlusPromega file contains all the transitions acquired.

## Technical Validation

### Assessing peptide precision using pooled CSF quality control (QC) standards

**Fig. 2:**
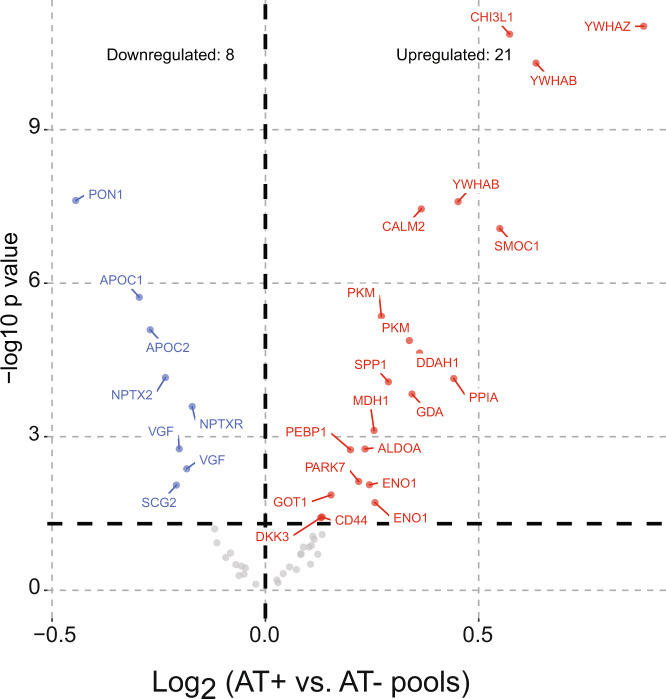
Differentially abundant peptides representing changed proteins in AT− vs AT+ QC CSF pools. The differentially abundant proteins in the QC pools were used to check the accuracy of the fold change consistent with our other studies^18^. We found 21 upregulated and 10 downregulated peptides. This result validated the direction of change of six proteins nominally significantly downregulated in previously published discovery proteomics (PON1, APOC1, NPTX2, VGF, NPTXR, and SCG2), and sixteen proteins previously reported as upregulated (YWHAZ, GDA, CHI3L1, PKM, CALM2, SMOC1, YWHAB, MDH1, ALDOA, ENO1, GOT1, PPIA, DDAH1, PEBP1, PARK7, and SPP1)^18,24^.

We generated two pools of CSF reference standards as QCs based on biomarker status (AT− and AT+). These QCs were processed and analyzed (at the beginning, end, and after every 20 samples per plate) identically to the individual clinical samples for testing assay reproducibility. We analyzed 30 QCs (15 AT− and 15 AT+) over approximately 5 days during the run of clinical samples. We identified 62 peptides from 51 proteins as reliably measured in the pooled reference standards. Notably, only 5 of these peptides overlap with the previously published PRM dataset given the unique differences in sample preparation, MS platform, and peptide selection^24^. We included 58 peptides from 51 proteins in our biomarker analysis, plus peptides specific for the four APOE alleles for proteogenomic confirmation of APOE genotypes^31,32^. The technical coefficient of variation (CV) of each peptide was calculated based on the peptide area ratio for the biomarker negative (AT−) and positive (AT+) QCs. We defined CSF peptide biomarkers with CVs ≤20% as quantified with high precision in these technical replicates which were un-depleted and unfractionated CSF sample pools. Technical and process reproducibility for all reported peptides was below 20% (CV <20%) in at least one pooled reference standard. The average CVs for all peptides in the AT− and AT+ QCs were 13% and 12%, respectively. The QC statistics for the biomarker and APOE allele-specific peptides are in the Data folder deposited on Sypnase^25^. Levels of HBA and HBB peptides can be used to assess potential blood contamination^33^ in each of the CSF samples. Correction for blood contamination could improve the statistics; however, no correction was performed for the statistical analyses presented. We used the protein directions of change to assess accuracy in the QC pools. The volcano plot between 54 peptides measured in the pools highlights peptide/protein levels that are consistent with previously reported AD biomarkers (Fig. 2)^18,24^. Albumin (2), hemoglobin (2), and APOE allele-specific (4) peptides are monitored in individual CSF samples for blood contamination or to confirm APOE genotype and were therefore removed from the pooled QC CSF volcano in Fig. 2.

### Monitoring LC-MS/MS instrument performance

**Fig. 3:**
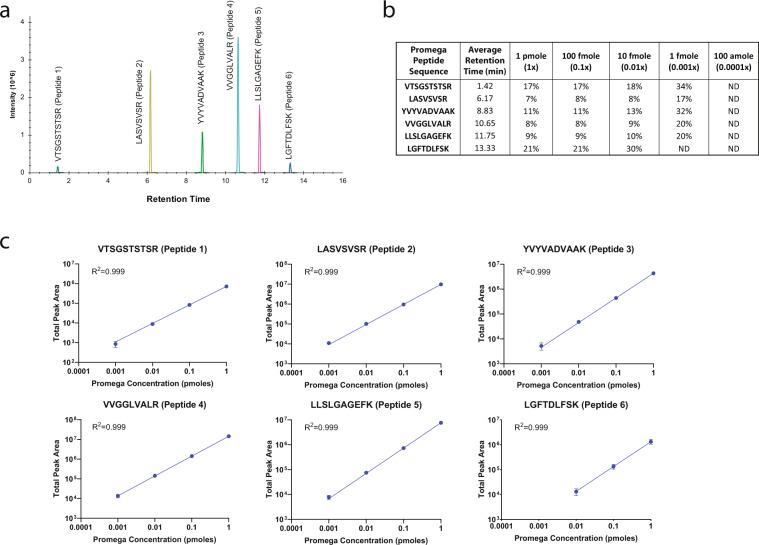
Isotopologue peptide internal reference standards to determine consistency of LC-MS/MS platform. Each of the CSF samples were spiked with a six-peptide, 5 isotopologue concentration LC-MS/MS Peptide Reference Mix from Promega (50 fmol/µL). (**a**) Extracted ion chromatogram for the 6 peptide (1pmol) mixture illustrating the wide range of retention times due to their hydrophobicity. (**b**) The raw peak areas in 423 injections over 5 days were used to determine the label-free CV for each peptide isotopologue estimating the lowest limits of detection to be between 1–10 fmole for each peptide. (**c**) The 5 unique isotopologues are used to assess the dynamic range across the gradient profile and each peptide demonstrates linearity across 3–4 orders of magnitude in the batch of 423 injections. Error bars represent the standard deviation across 423 injections.

The sample reconstitution solution contained Promega 6 × 5 LC-MS/MS Peptide Reference Mix (50 fmol/µL)^34^. The Promega Peptide Reference Mix provides a convenient way to assess LC column performance and MS instrument parameters, including sensitivity and dynamic range. The mix consists of 30 peptides; 6 sets of 5 isotopologues of the same peptide sequence, differing only in the number of stable, heavy-labeled amino acids incorporated into the sequence using uniform ^13^C and ^15^N atoms making them chromatographically indistinguishable. The isotopologues were specifically synthesized to cover a wide hydrophobicity range so that the dynamic range could be assessed across the gradient profile (Fig. 3a). Each isotopologue represents a series of 10-fold dilutions, estimated to be 1 pmole, 100 fmole, 10 fmole, 1 fmole, and 100 amole for each peptide sequence in a 20 µL injection, a range that would challenge the lowest limits of detection of the method (Fig. 3b). We assessed the raw peak areas in 423 injections over 5 days to determine the label-free CV for each peptide isotopologue (Fig. 3b). The 100-amole level (0.0001x) was not detected (ND) for any of the peptide sequences. Based on the label-free CV, we determined the lowest limit of detection for each peptide to be between 1–10 fmole across the gradient profile with a dynamic range spanning 4 orders of magnitude for all peptides except the latest eluting peptide at 13.3 minutes (Fig. 3c).

### Technical replicate variance

**Fig. 4:**
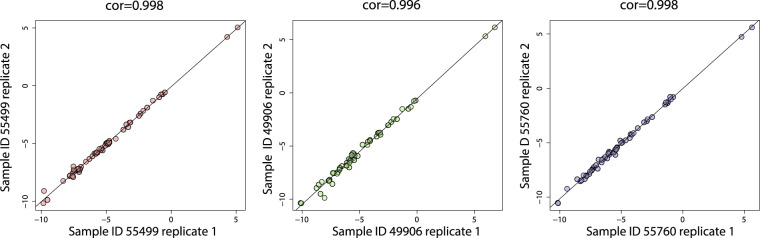
Technical reproducibility of peptide measurements in replicate CSF samples. Pearson correlation and p-value of replicate measures of 58 peptides in the 3 replicated CSF samples that were analyzed randomly within the series of 423 injections by SRM-MS.

Three individual samples were analyzed in duplicate scattered throughout the sample run sequence to assess technical replicate variance. We graphed the log2(ratio) for each of 58 biomarker peptides in replicate 1 (x-axis) versus replicate 2 (y-axis) for each sample and determined the Pearson correlation coefficient with associated P value (Fig. 4). The analysis showed a near-identical correlation (ρ = 0.996–0.998; p < 1e-200) between each of the technical replicate pairs for the three individual CSF samples, supporting the same high level of method reproducibility we found using the QC pools.

### Concordance between a discovery (ssTMT) and replication (SRM) datasets

**Fig. 5:**
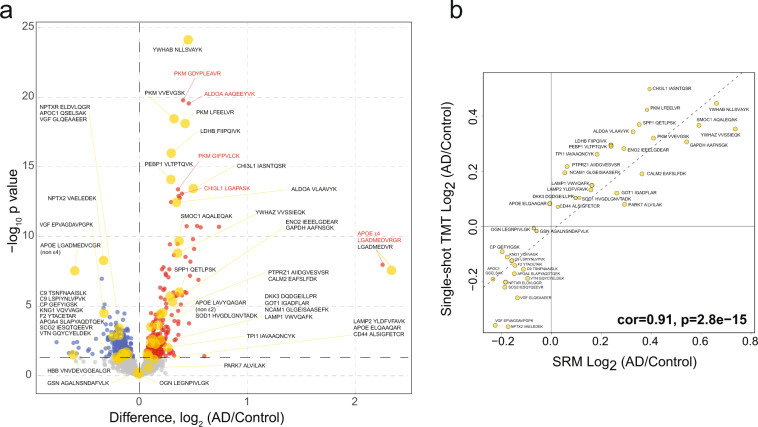
Peptide concordance between SRM and ssTMT datasets. (**a**) Volcano plot displaying the log2 fold change (FC) (x-axis) against t-test log10 p-value (y-axis) for all peptides (n = 2,340) comparing AD (n = 150) versus Controls (n = 147). Cutoffs were determined by significant differential expression (p < 0.05) between control and AD cases. Peptides with significantly decreased levels in AD are shown in blue while peptides with significantly increased levels in disease were indicated in red. 44 of 62 SRM peptides that overlap with this ssTMT dataset and SRM are highlighted as larger yellow points with black text labels. Red text and traces to red points are labels for peptides not included in the current SRM study that were significantly upregulated in the ssTMT dataset. (**b**) Correlation between the fold-change (AD vs control) of all selected overlapping peptides (n = 40) across SRM (x-axis) and ssTMT (y-axis) were strongly correlated (cor = 0.91, p = 2.8e−15).

Since our peptide targets were largely based on multiple single-shot tandem mass tag (ssTMT) datasets^18^, we generated a representative ssTMT peptide level volcano from one of these datasets comprised of 297 individuals (147 control and 150 AD) (Fig. 5a). There are 44 of 62 SRM peptides that overlap with this ssTMT dataset and are highlighted in yellow on the volcano plot (Fig. 5a). To establish peptide concordance, we also compared the direction of change or effect size (log2 fold change) for 40 overlapping peptides, excluding albumin, hemoglobin, and APOE allele-specific peptides. Figure 5b shows significant correlation (cor = 0.91; p = 2.8e-15) between SRM and ssTMT peptide highlighting the accuracy and concordance of measurements across both MS assays. Thus, despite substantial differences in chromatography (nanoflow versus standard flow), MS instrumentation (Orbitrap versus triple quadrupole), and protein quantitation approaches (ssTMT versus SRM), the selected peptides in this assay are highly reproducible and robust in their direction of change in AD CSF. Furthermore, the enhanced throughput of the SRM protocol (96 samples per day) allows us to examine large cohorts relatively quickly as compared to previously published unbiased discovery proteomics^35^ and parallel reaction monitoring^24^ experiments.

### Stage-specific differences in peptide and protein levels

**Fig. 6:**
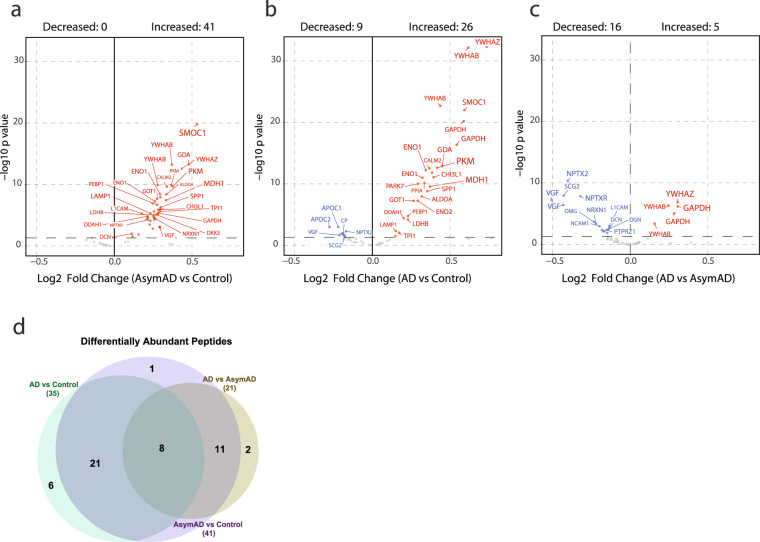
Differential expression analysis across stages of AD. ANOVA analysis with Tukey *post hoc* FDR was performed for pairwise comparison of mean log2(ratio) differences between the 3 stages of AD (i.e., Control, AsymAD and AD) of N = 390 total case samples and plotted as a volcano. Significance threshold for counting of peptides was p < 0.05 (dashed horizontal line). Differentially expressed peptides for (**a**) AsymAD (N = 127) versus control (N = 133), (**b**) AD (N = 130) versus control, and (**c**) AD versus AsymAD are labeled by their gene symbols. (**d**) Counts of peptides with significant difference in any of the 3 dichotomous comparisons are presented as a Venn diagram. Full statistics from the ANOVA and Tukey post-hoc analysis is presented in the ANOVA table in the Data folder deposited on Synapse^25^.

The described cohort includes control, AsymAD, and AD groups across the Amyloid/Tau/Neurodegeneration (AT/N) framework^36^, which allows for the comparison of peptide and protein differential abundance across stages of disease. By comparing candidate biomarkers using ANOVA (excluding APOE allele-specific peptides), we found 41 differentially expressed peptides (36 proteins) in AsymAD vs controls (Fig. 6a), 35 differentially expressed peptides (30 proteins) in AD versus controls (Fig. 6b), and 21 differentially expressed peptides (18 proteins) in AD vs AsymAD (Fig. 6c). These changes are consistent to previous proteomic studies of AD CSF^18,24,37,38^. The Venn diagram summarizes the differentially expressed peptides across groups in Fig. 6d.

**Fig. 7:**
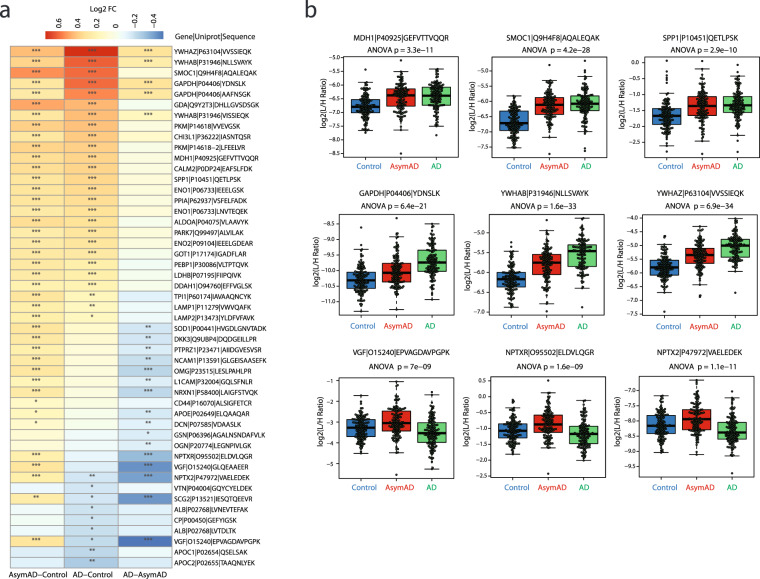
Stratifying early from progressive biomarkers of AD. (**a**) The magnitude of positive (red) and negative (blue) changes are shown on a gradient color scale heatmap representing mean log2-fold change (Log2 FC) for each of 49 peptides significant in any of the 3 group comparisons. Tukey significance of the pairwise comparisons is indicated by overlain asterisks; *p < 0.05, **p < 0.01, ***p < 0.001. (**b**) Peptide abundance levels of selected panel markers that are differentially expressed between groups. The upper row highlights biomarkers that are significantly different in AsymAD versus controls, but not significantly different in AsymAD versus AD. The middle row of 3 peptides highlights progressive biomarkers of AD, which show a stepwise increase in abundance from control to AsymAD to AD cases. The bottom row highlights a set of proteins that are increased in AsymAD compared to controls but decreased in AD versus control or AsymAD samples.

Using a differential abundance analysis, we were able to stratify the changing proteins as early or progressive biomarkers of AD (Figs. 6, 7). The log_2_-fold change (Log2 FC) from the volcano plots in Fig. 6 are represented as a heatmap in Fig. 7a to illustrate how each peptide is changing across each group comparison. Twenty-two peptides (21 proteins) were early biomarkers of AD because they were significantly different in AsymAD versus controls, but not significantly different in AD versus AsymAD (Fig. 7a). A plurality of these proteins mapped to metabolic enzymes linked to glucose metabolism (PKM, MDH1, ENO1, ALDOA, ENO2, LDHB, and TPI1)^15,16,24^. SMOC1 and SPP1, markers linked to glial biology and inflammation^16,18,24^, were also increased in AsymAD samples compared to controls (Fig. 7b, top *row*). GAPDH, YWHAB and YWHAZ proteins were found to be progressive biomarkers of AD because the proteins were differentially expressed from Control to AsymAD and from AsymAD to AD with a consistent trend in direction of change (Fig. 7b, *middle row*)^18^. Proteins associated with neuronal/synaptic markers including VGF, NPTX2, NPTXR, and L1CAM were increased in AsymAD compared to controls but decreased in AD vs controls (Fig. 7b, l*ower row*)^18,24^. Interestingly, we found 14 peptides (13 proteins) that were up in AsymAD as compared to Control but down in AD when compared to AsymAD. A majority of these proteins map to neuronal/synaptic markers including VGF, NPTX2, NPTXR, which are some of the most correlated proteins in post-mortem brain to an individual’s slope of cognitive trajectory in life (Fig. 7a,b, l*ower row*)^39^. The peptide ANOVA analysis data and corresponding modules^40^ and/or panels^18^ are located in the ANOVA file in the Data folder deposited on Synapse^25^.

### Correlation of peptide biomarker abundance to Aβ(1–42), Tau, pTau, and cognitive measures

**Fig. 8:**
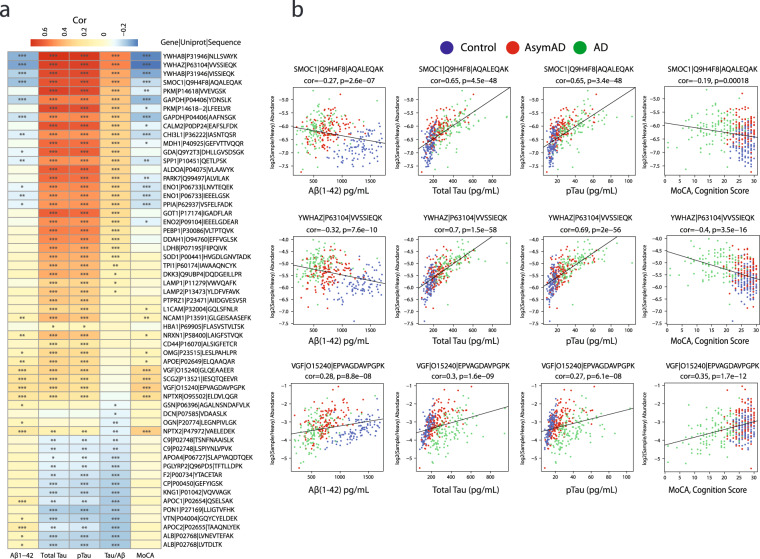
Correlating CSF peptide biomarker abundances to amyloid, Tau, and cognitive measures. (**a**) Positive (red) and negative (blue) Pearson correlations between biomarker peptide abundance and immunoassay measures of Aβ(1–42), total Tau, phospho-T181 Tau (pTau), ratio of total Tau/Aβ, and cognition (MoCA score). Student’s *t* test significance is indicated by overlain asterisks; *p < 0.05, **p < 0.01, ***p < 0.001. (**b**) Individual correlation scatterplots are shown for SMOC1 (upper row), YWHAZ (middle row), and VGF (lower row). Individual cases are colored by their diagnosis; blue for controls, red for AsymAD cases, and green for AD cases. Amyloid immunoassay measures of 1,700 (maximum, saturated value in the assay) were not considered for correlation. Pearson correlations (rho), Student p values of correlation significance, and numbers of paired observations for correlation of biomarker peptide abundances to immunoassay measures of Aβ(1–42), total Tau, phospho-T181 Tau, and the ratio of total Tau/ Aβ are in the ELISA_PearsonCors file in the Data folder on Synapse^25^.

The comparison of existing biomarkers to the SRM peptide measurements can be accomplished by correlation, where the degree of correlation indicates how similar a peptide measurement is to the established immunoassay-measured biomarkers of Aβ(1–42), total Tau, and pTau as well as cognition (MoCA score). In Fig. 8a, we demonstrate that 57 of the 58 biomarker peptides have significant correlation to at least one of the above biomarkers, or the ratio of total Tau/Aβ. Individual correlation scatterplots and linear fit lines for three of the peptides (SMOC1: AQALEQAK, YWHAZ: VVSSIEQK, and VGF: EPVAGDAVPGPK) are provided in Fig. 8b. Significant correlations of these peptides to the established biomarker and cognitive measures indicate the potential of these measurements to classify or stage disease progression. The targeted SRM measurement correlations largely agree with those observed from unbiased discovery proteomics^35^ and parallel reaction monitoring^24^ experiments.

### Receiver-operating characteristic (ROC) curves for evaluating biomarker diagnostic capability

**Fig. 9:**
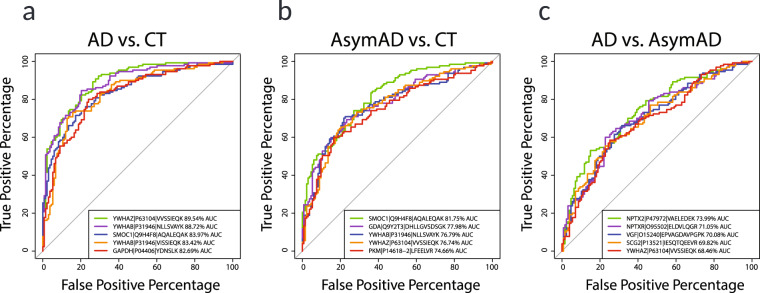
Receiver-operating characteristic (ROC) curve analysis of peptide diagnostic potential. ROC curves for each of three pairs of diagnosed case groups were generated to determine the top-ranked diagnostic biomarker peptides among the 58-peptide panel plus 4 APOE specific peptides. (**a**) A total of 263 AD (N = 130) and control (N = 133) CSF case samples were classified according to the logistic fit for each peptide’s log2(ratio) measurements across these samples, and the top 5 ranked by AUC are shown. (**b**) Top five performing peptides for discerning AsymAD (N = 127) from control (N = 133) case diagnosis groups are provided with AUCs, nominating these peptides as potential markers of pre-symptomatic disease, and as cognates for AT+ biomarker positivity. (**d**) Symptomatic AD (N = 130) and AsymAD (N = 127) discerning peptides were ranked by AUC and the top five ROC curves are shown and nominated as cognate CSF measures for compromised patient cognition. ROC curve statistics including AUC, p, 95% DeLong confidence interval, accuracy, specificity, and sensitivity for dichotomous diagnosis case sample groups are in the ROCstatistics file in the Data folder on Synapse^25^.

The capacity for peptide measurements to serve as a diagnostic biomarker distinguishing individuals with AD and even asymptomatic disease from individuals not on a trajectory to develop AD is well-established, with secreted amyloid and tau peptide measurements in CSF being the current gold standard for interrogation of patients’ AD stage from their CSF^41^ where CSF Aβ(1–42) concentration inversely correlates to plaque deposition in the living brain^42^. The measurements of additional peptides collected here are appropriate for comparison to immunoassay measurements of CSF amyloid and Tau biomarker positivity, or a dichotomized cognition rating, or other ancillary traits such as diagnosis for the 390 individuals. To demonstrate this utility, we performed receiver-operating characteristic (ROC) curve analysis and calculated the area under the curve (AUC) for all 62 peptide measures as fitting a logistic regression to 3 subsets of samples divided to represent known pairs of disease stages, namely AD versus control, AsymAD versus control, and AD vs AsymAD (Fig. 9). The top performing peptide for the YWHAZ gene product 14-3-3 ζ protein demonstrated an AUC of 89.5% discrimination of AD from control cases consistent with previous studies^24,37,43^. SMOC1 AUC of 81.8% was the best performing peptide for discrimination of AsymAD from control groups^24^. In contrast, the synaptic peptides to NPTX2 (AUC of 74.0%), NPTXR (AUC of 71.1%), VGF (AUC of 70.1%) and SCG2 (AUC of 69.8%) best discriminated AD from AsymAD groups suggesting that neurodegeneration due to AD pathology is occurring in the symptomatic phase of disease^44^. Figure 9 shows the top five peptides by AUC for each of the three comparisons, highlighting the potential of this data set to aid in the design or validation of stage-specific biomarkers. Additional future analysis using these peptides alone or in combination could be used to subtype, predict disease onset, and gauge treatment efficacy.

## Usage Notes

This targeted mass spectrometry dataset serves as a valuable resource for a variety of research endeavors including, but not limited to, the following applications:

### Use case 1: Peptide abundance in CSF

This dataset provides a reference for peptide detectability in CSF under relatively high-throughput conditions, especially if an investigator wants to determine whether their protein of interest has abundance above the lower limit of detection in CSF under these analytical conditions. Raw data deposited on Synapse^25^ contains transitions for over 200 peptides that were robustly detected in CSF discovery proteomics^18,24^.

### Use case 2: Confirmation of APOE allele genotyping

Apolipoprotein E (ApoE) has three major genetic variants (E2, E3, and E4, encoded by the ε2, ε3 and ε4 alleles, respectively) that differ by single amino acid substitutions^45^. APOE genotype is closely related to AD risk^46^ with ApoE4 having the highest risk, ApoE2 the lowest risk, and ApoE3 with intermediate risk^47,48^. Due to the amino acid substitutions in each variant, there are allele-specific peptides that can be targeted by mass spectrometry^31,49^. We monitored CLAVYQAGAR (APOE2), LGADMEDVR (APOE4), LGADMEDVCGR (APOE2 or APOE3), and LAVYQAGAR (APOE3 or APOE4) to confirm the APOE genotype of each CSF sample in a concurrent SRM-MS method^25^. The CV for each APOE peptide in each QC is listed in the QCstatistics file in the Data folder on Synapse^25^. Previous studies report the association of APOE genotype with various clinical, neuroimaging, and biomarker measures^50–53^. Exploring the relationship between APOE status and the CSF biomarker peptides presented requires further analysis reserved for future studies.

## Data Availability

Custom code generating figures and tables including correlation plots, volcanoes, Venn diagram, annotated heatmap, statistics tables, and ROC curves is available for download with registration for a free account on synapse.org. The code is available as R scripts in the Analysis folder deposited on Synapse^25^. These scripts were run as provided on R version 4.0.2 with the two provided input files to generate outputs.
